# Characterizing health state utilities associated with Duchenne muscular dystrophy: a systematic review

**DOI:** 10.1007/s11136-019-02355-x

**Published:** 2019-12-06

**Authors:** Shelagh M. Szabo, Ivana F. Audhya, Daniel C. Malone, David Feeny, Katherine L. Gooch

**Affiliations:** 1 Broadstreet HEOR, 203 – 343 Railway St, Vancouver, BC Canada; 2 Sarepta Therapeutics Inc., 215 First St, Cambridge, MA 02142 USA; 3 College of Pharmacy, The University of Arizona, Tucson, AZ USA; 4 McMaster University, Hamilton, ON Canada

**Keywords:** Duchenne muscular dystrophy, DMD, Utility values, Preferences, Health state, Systematic review

## Abstract

**Background:**

Preferences for health states for Duchenne muscular dystrophy (DMD) are necessary to assess costs and benefits of novel therapies. Because DMD progression begins in childhood, the impact of DMD on health-related quality-of-life (HRQoL) affects preferences of both DMD patients and their families. The objective of this review was to synthesize published evidence for health state utility from the DMD patient and caregiver perspectives.

**Methods:**

A systematic review was performed using MEDLINE and Embase, according to best practices. Data were extracted from studies reporting DMD patient or caregiver utilities; these included study and patient characteristics, health states considered, and utility estimates. Quality appraisal of studies was performed.

**Results:**

From 888 abstracts, eight publications describing five studies were identified. DMD utility estimates were from preference-based measures presented stratified by ambulatory status, ventilation, and age. Patient (or patient–proxy) utility estimates ranged from 0.75 (early ambulatory DMD) to 0.05 (day-and-night ventilation). Caregiver utilities ranged from 0.87 (for caregivers of adults with DMD) to 0.71 (for caregivers of predominantly childhood patients). Both patient and caregiver utilities trended lower with higher disease severity. Variability in utilities was observed based on instrument, respondent type, and country. Utility estimates for health states within non-ambulatory DMD are under reported; nor were utilities for DMD-related health states such as scoliosis or preserved upper limb function identified.

**Conclusion:**

Published health state utilities document the substantial HRQoL impacts of DMD, particularly with disease progression. Additional research in patient utilities for additional health states, particularly in non-ambulatory DMD patients, is warranted.

**Electronic supplementary material:**

The online version of this article (10.1007/s11136-019-02355-x) contains supplementary material, which is available to authorized users.

## Introduction

Duchenne muscular dystrophy (DMD) is a rare X-linked severe progressive myopathy caused by mutations in the gene for dystrophin, with an estimated birth prevalence of approximately 1:5000 males [1, 2]. The typical phenotype includes progressive muscle weakness in childhood and loss of ambulation early in the second decade of life [3]. The development of cardiomyopathy and respiratory insufficiency in the teens to early 20 s contributes to need for ventilation support and reduced life expectancy [4]. The current standard of care for DMD is treatment with corticosteroids to slow disease progression [5]. Newer antisense oligonucleotides and emerging gene therapy treatments have the potential to modify the disease course for patients with DMD [6].

Access to any new treatments requires consideration of their cost and benefit profile versus existing therapies, and cost-effectiveness analyses are frequently conducted to determine their “value” for reimbursement decision-making. As part of cost–utility evaluations in particular, assessing the impact of a disease on health-related quality-of-life (HRQoL) is required. *Health state utility values* represent the strength of individuals’ preferences for specific health states or conditions (e.g. how good or bad a person thinks the health state is), using a scale typically anchored at 1 (full health) and 0 (dead) [7]. Utility values are then used to estimate quality-adjusted life years in economic models [8]. A variety of methods exist by which to elicit utility values. These include both direct elicitation methods [such as the time trade-off (TTO), standard gamble (SG), or visual analogue scale (VAS)], as well as indirect methods using generic preference-weighted HRQoL measures such as the EuroQol five dimensions questionnaire (EQ-5D) or the Health Utilities Index (HUI) [9–11]. The indirect multi-attribute health status classification systems assign respondents to a set of non-disease-specific health states based on an individual’s combination of responses provided to a series of questions that measure different attributes of health. Such indirect preference-weighted measures have been widely recommended for use in economic evaluations in part because they incorporate the societal perspective in that the scoring functions for these measures are based on preference scores obtained from representative samples of the general population. Applying these weights generates utility estimates that allow comparison across healthcare interventions and sectors [12–14]. While many disease-specific measures exist that may better capture the HRQoL impact of a condition like DMD, algorithms to convert these scale scores to utilities are limited [15].

In recent years, a number of publications describing utility values for DMD health states have been published [16, 17]. However, how utility values for health states compare across studies, how patient characteristics and other key factors influence estimated utility, and which health states have been considered, has not been synthesized. Nor has the impact of DMD on caregiver utility been synthesized which is important as there is also a considerable caregiver and family burden associated with DMD [18]. The objective of this study was to review, synthesize, and appraise existing evidence for utility values for DMD health states from the perspectives of both patients and their caregivers.

## Methods

A systematic literature review (SLR) was performed to identify and critically appraise published evidence on utility values for health states describing the HRQoL impact of DMD, for both patients and caregivers.

### Search strategy and study selection

A comprehensive search strategy was implemented in Medline and EMBASE to identify eligible records published from database inception to January 11, 2019 (see Supplementary Table 1). The search terms included DMD and the following concepts: cost-effectiveness; utilities; preferences; health states; and HRQoL; and a variety of potential respondent types (patient, caregiver, physician).The inclusion and exclusion of studies in the SLR was guided by PICOS (Population, Intervention, Comparator, Outcomes, Study design) criteria (Table 1), developed following the Preferred Reporting Items for Systematic Reviews and Meta-Analysis (PRISMA) guidelines [19]. Briefly, articles describing directly- or indirectly-elicited utilities for DMD health states, from patients, physicians, caregivers or members of the general public were eligible for inclusion. Citations were de-duplicated prior to double abstract screening and full-text review being performed to identify the subset of eligible articles for data extraction. To identify additional eligible articles the following were also evaluated: (1) published economic models that were identified by the search strategy, for the source of their utility estimates; (2) the Cost-effectiveness Analysis Registry at Tufts Medical Center [20]; and (3) the citations of included studies. In addition to searching for published manuscripts, meeting abstracts listed in Medline (in the ‘in process’ database) and EMBASE from the most recent two years were also screened.

**Table 1:** PICOS criteria to define the scope of the literature review

Category	Criteria
Population to provide valuation of DMD health states	Patients with DMD; or parents/caregivers who provide responses on their behalf (e.g. ‘proxies’) Clinicians who manage patients DMD ‘Layperson respondents’: Individuals who represent the general population^a^ Caregivers of DMD patients
Intervention/comparators	None
Outcomes	Directly-elicited utilities/preference values, e.g.
	Standard gamble
	Time trade-off
	Indirectly-elicited utilities/preference values, e.g. using the
	EQ-5D
	HUI-3
	SF-6D
Study design	Prospective or retrospective studies Clinical trials

Case reports or case series, animal studies, and articles not in English were excluded

*DMD* Duchenne muscular dystrophy, *EQ-5D* Euro-QoL 5-dimension survey, *SF-6D* Short-form 6-D, *HUI-3* Health Utilities Index Mark 3

^a^Layperson respondents were considered, in case for example, vignette-based exercises using members of the general population were identified as sources of utility estimates

### Data extraction

Data from eligible studies were extracted by two independent reviewers and recorded in Microsoft Excel^®^, with discrepancies resolved through discussion to achieve consensus. The following data were extracted from each study: study author and year of publication, study design, geographic location, baseline clinical and demographic characteristics, sample size, instrument, respondent type (patient, parent/caregiver; and proxy or direct), and utilities estimates. For continuous variables, the mean, median, standard deviation (SD), and range were extracted where available. For dichotomous and categorical variables, the number of patients and proportion were extracted.

### Data synthesis and quality appraisal

Mean utility values, and the number of individuals contributing to each estimate, were summarized according to health state: overall, and by clinically important subgroup (e.g. by ambulatory status or need for ventilation). Mean utility values were stratified by respondent type, country, and by patient physical or mental health (as presented by the original investigators).

To our knowledge, there are no agreed-upon reporting standards for studies describing health state preference studies. A critical appraisal of the quality of the studies contributing estimates was therefore performed using a previously-published framework described by Papaionannou et al. [21] Within the study quality assessment, one point was awarded for each of the following criteria: (1) sample size ≥ 100; (2) description of respondent selection and recruitment; (3) description of inclusion/exclusion criteria; (4) response rate ≥ 60%; (5) reporting of attrition/loss to follow-up (for longitudinal studies only); (6) reporting of missingness of data and approaches to deal with it; (7) appropriateness of measure (based on the review authors’ judgment). Lastly, the scores were summed for each article to yield an overall quality score, ranging from 0 to 7 (for longitudinal studies) or 0 to 6 (for cross-sectional studies) where higher scores indicated higher quality [22].

## Results

### Study selection

**Fig. 1:**
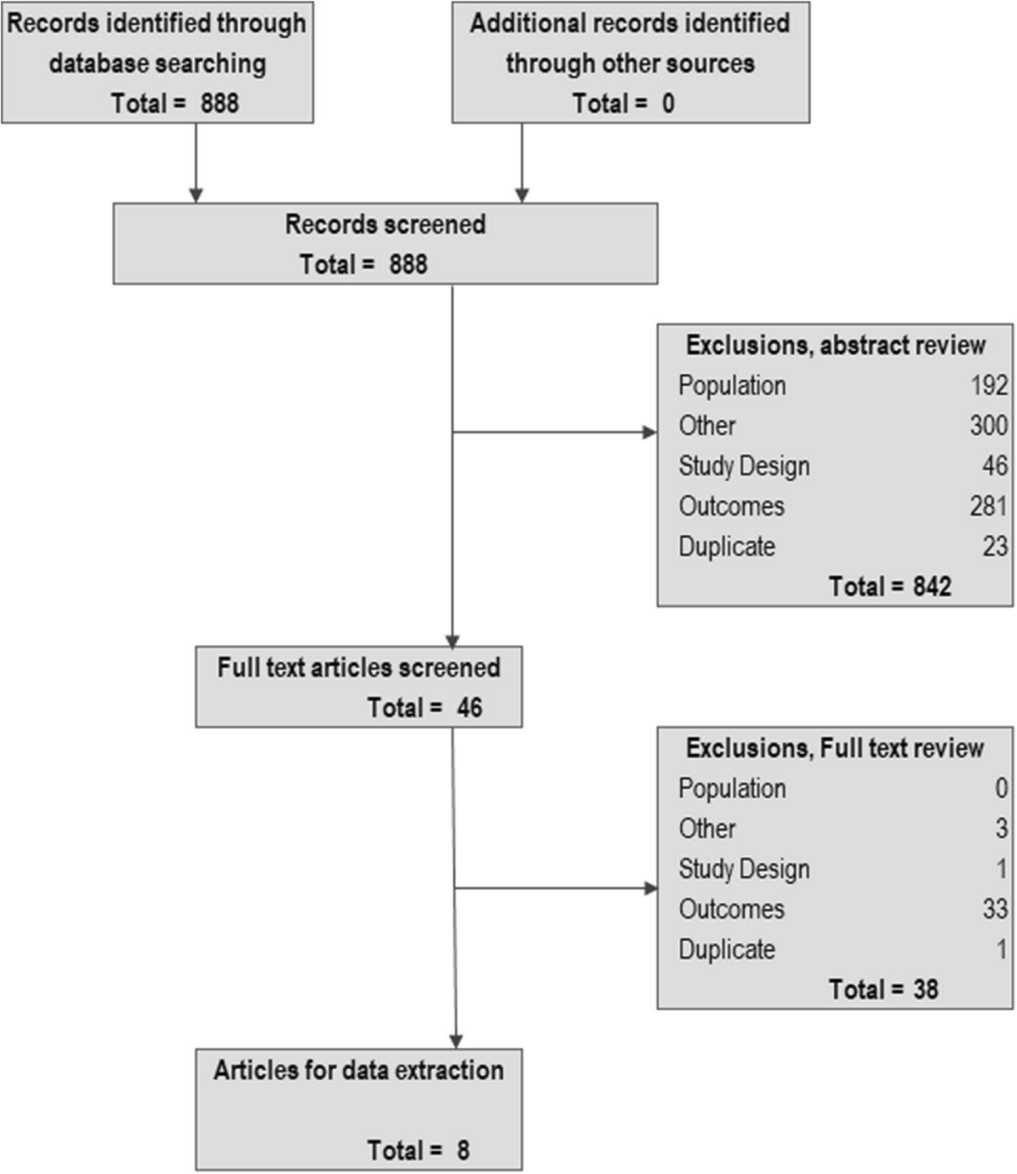
PRISMA diagram. Footnote: ‘Other sources’ would include additional relevant articles identified from hand-searching reference lists, as inputs of economic models, or from the Cost-effectiveness Analysis Registry at Tufts Medical Center. Note, no additional articles were identified from any of these sources

Implementing the search strategy identified 888 potentially relevant studies, 842 of which (94.8%) were excluded on abstract review (see PRISMA diagram, Fig. 1). Of the remaining 46 articles, 38 were excluded on full-text review; most (*n* = 33; 87%) because they did not present utility values, with the remainder being excluded for study design (*n* = 1), being a duplicate (*n* = 1), or other reasons (in this case, excluded due to publication type, *n* = 3). The remaining eight full-text reports were identified for inclusion, two of which reported on the same *patient* study sample and one a sub-sample of those patients; and two of which reported on the same *caregiver* study sample and one a sub-sample of those caregivers [15, 17, 18, 23]. This resulted in five unique studies or samples, where one set of caregiver-patient dyads was represented in four publications (Table 2) [15, 17, 18, 23–27]. No additional records were identified from the Tufts database or examining article references.

**Table 2:** Characteristics of studies, and their respondents, included in the systematic review of DMD utilities

Citation	Study design	Objective	Countries	Data source	Respondents	Mean (range or *SD*) age (y)	Utility measure
Cavazza et al. [24]	Cross-sectional	HRQoL and economic burden of DMD in Europe	Bulgaria, France, Germany, Hungary, Italy, Spain, Sweden, UK	Parent Project MD	Community-dwelling males with DMD	14.7 (11.3–23.9)^b^	EQ-5D utility and VAS
					Caregivers of community-dwelling DMD patients	44.3 (25.0–49.6)^b^	EQ-5D utility and VAS
17^c^ [17]	Cross-sectional	Economic burden of DMD	Germany, Italy, UK, US	TREAT-NMD	Males with DMD aged ≥ 5 years	14 (8–17)	HUI-3
					Caregivers of males with DMD aged ≥ 5 years	44 (39–50)	EQ-5D utility
Landfeldt et al. [23]^c^	Cross-sectional	HRQoL impact of DMD	Germany, Italy, UK, US	TREAT-NMD	Males with DMD aged ≥ 5 years	14 (8–17)	HUI-3
					Males with DMD on ventilatory support	NR	HUI-3
Landfeldt et al. [18]^c^	Cross-sectional	Burden among caregivers of patients with DMD	Germany, Italy, UK, US	TREAT-NMD	Caregivers of males with DMD aged ≥ 5 years	44 (39–50)	EQ-5D utility and VAS
Landfeldt et al. [25]	Cross-sectional	Psychometric properties of PedsQL NMM	UK, US	TREAT-NMD	DMD patients who could complete the PedsQL NMM	16 (*7*)	HUI-3
Magnetta et al. [26]^a^	N/A	Cost-effectiveness model for treatment for advanced HF in DMD		NA	Hypothetical cohort of patients with DMD and advanced HF	NA	EQ-5D utility
Pangalila et al. [27]	Cross-sectional	Burden among caregivers of **adult** patients with DMD	Netherlands	NA	Patients with DMD aged ≥ 20 years	27 (*6.1*)	EQ-5D utility
					Caregivers of patients with DMD aged ≥ 20 years	57 (*6.8*)	EQ-5D utility
Landfeldt et al. [15]^c^	Cross-sectional	Psychometric properties of the DMDSAT	UK	TREAT-NMD	Males with DMD aged ≥ 5 years	14 (5–43)	HUI-3
					Caregivers of males with DMD aged ≥ 5 years	NR	EQ-5D

Intraquartile range was not reported

*HRQoL* health-related quality of life, *DMD* Duchenne muscular dystrophy, *HF* heart failure, *UK* United Kingdom, *US* United States, *SD* standard deviation, *NR* not reported, *NA* not applicable, *HUI* Health Utilities Index, *EQ-5D* EuroQoL 5 dimensions, *VAS* visual analogue scale, *MD* muscular dystrophy, *NMD* Neuromuscular dystrophy

^a^Utilities were not directly collected in Magnetta et al. but based on a previous data collection exercise that used the EQ-5D

^b^The mean age for the overall sample was 14.7 years; and the range reflects the mean age of each country-specific sample contributing to the overall estimate

^c^Four publications from the same related sample/overall study; Landfeldt (2015) focused on the UK subset only

### Study characteristics

All identified studies used indirect elicitation methods, specifically the EQ-5D-3L (*n* = 4; 2 of which also reported the EQ-5D VAS) [15, 17, 18, 24, 26, 27] and HUI-3 (*n* = 2) [18, 23, 25]. One study estimated DMD-specific cardiomyopathy utilities for an economic model [26] by down-weighting [28] existing trial-based EQ-5D utility values collected from patients treated with eplerenone for heart failure after acute myocardial infarction. The other four studies were cross-sectional and recruited patients from Bulgaria, France, Germany, Hungary, Italy, Spain, Sweden, the United Kingdom (UK), the United States (US), and the Netherlands. One of those studies used utility data from UK-based respondents and mapped responses to the Duchenne Muscular Dystrophy Functional Ability Self-Assessment Tool (DMD SAT) [15]. As only cross-sectional studies were identified, no data on change in utilities over time were available.

Utilities for patient health states were available from all five studies; in addition to the aforementioned economic model [26], three studies used direct patient report [15, 24, 25, 27] and one study used caregiver-proxy report [15, 17, 23]. Mean patient ages ranged from 14 to 27 years. Caregiver utilities were available from three studies [15, 17, 18, 24, 27]. Except for the economic model that presented insufficient data to gauge the quality of the utilities elicitation, the quality of all studies were rated between 4 and 5 out of 6 (Supplementary Table 2).

### Patient utility estimates

**Fig. 2:**
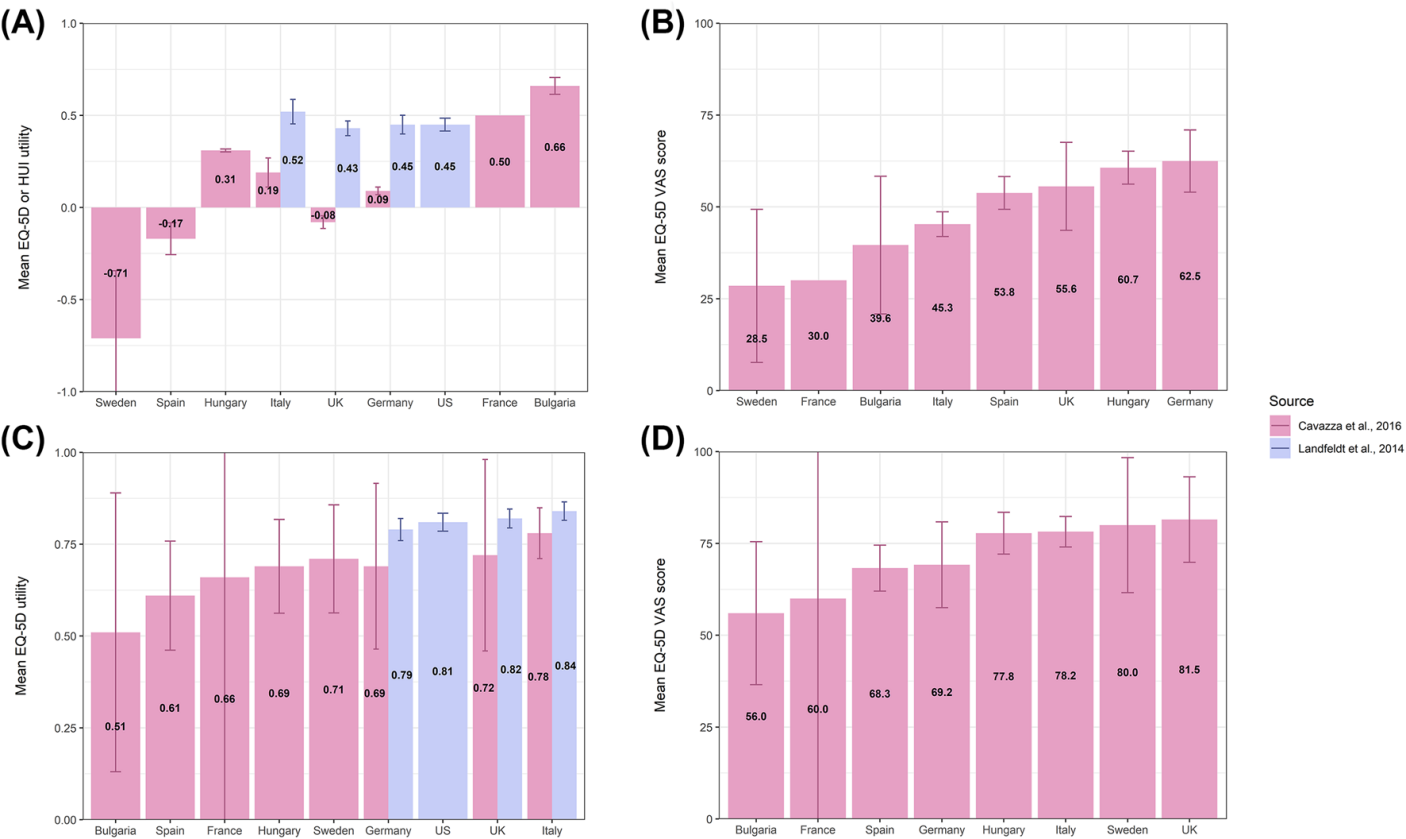
Country-specific DMD patient utilities **a** by HUI (Landfeldt et al.), EQ-5D (Cavazza et al.), **b** or EQ-5D VAS; and DMD caregiver utilities by **c** EQ-5D or **d** EQ-5D VAS. Footnote: See Tables 1 and 2 for descriptions of overall patient populations by study

**Fig. 3:**
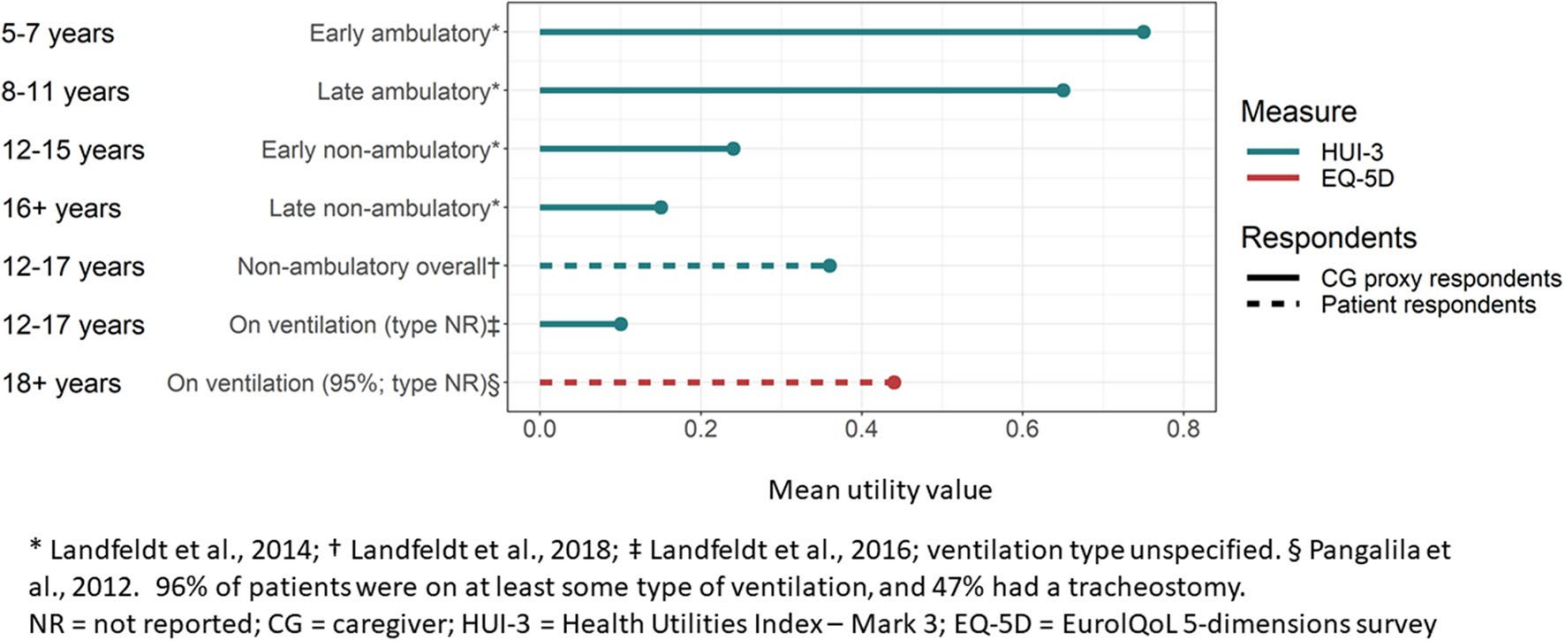
DMD patient health state utilities by age and ambulatory/respiratory status, according to respondent type (patient vs. proxy) and measure (HUI-3 and EQ-5D)

Patient utility estimates were derived from samples with DMD of mixed ages and functional statuses, and are presented in Table 3 and Supplementary Table 3. Study groups included ambulatory and non-ambulatory patients, patients with or without ventilatory support, and patients with cardiomyopathy. Utility estimates ranged from a patient-based EQ-5D utility of 0.24 (EQ-5D VAS 50.5) [24] to a caregiver-proxy HUI-3 utility of 0.46. HUI-3 utilities from that study, stratified by ambulatory status and age, ranged from 0.75 (for patients in the early ambulatory stage; age 5-7 years) to 0.15 (for patients in the late non-ambulatory stage; age 16 + years) [17, 23]. In a psychometric validation study of the PedsQL NMM instrument, the mean (SD) patient-based HUI-3 utility for non-ambulatory DMD was 0.36 (0.28) [25]. One caregiver-proxy-based HUI-3 utility for needing ventilation was identified (0.1) [23] and estimates from the UK subset of that study ranged from 0.05 (for night- and day-time ventilation) to 0.52 (for DMD patients not requiring ventilation) [15]. Both utility estimates for needing ventilatory support from Landfeldt were lower than a patient-based EQ-5D estimate for needing ventilatory support (0.44) from a Dutch study of adults with DMD [27]. Considerably more intra-country variability was observed from the EQ-5D-based utilities from Cavazza et al. [24], compared to the HUI-3-based utilities from Landfeldt et al. [17], although country-specific samples in the former study were small (Fig. 2). Estimated utilities for DMD-related cardiomyopathy are provided in Supplementary Table 3, and DMD utilities plotted by age are presented in Fig. 3. No utilities for other DMD-related health states (for example scoliosis, upper limb function or developmental disability) were identified; nor were utility values reported for any subgroups of patients with non-ambulatory DMD, except for those on ventilatory support.

**Table 3:** Utilities for clinical stages of DMD

Health state		Patient respondents		CG proxy respondents		
	*n*	Measure	Mean (SD) utility	Measure	Mean (SD) utility	Source
Ambulatory						
Early ambulatory (age 5–7 years)	155			HUI-3	0.75	17^a^ [17]
Late ambulatory (age 8–11 years)	256			HUI-3	0.65	17^a^ [17]
Any ambulatory	411			HUI-3	0.69	Estimated from [17]
Non-ambulatory						
Early non-ambulatory (age 12–15 years)	154			HUI-3	0.24	17^a^ [17]
Late non-ambulatory (age 16 + years)	205			HUI-3	0.15	17^a^ [17]
Overall; age not specified	278	HUI-3	0.36 (0.28)			Landfeldt et al. [25]
Any non-ambulatory	359			HUI-3	0.19	Estimated from 17 [17]
On ventilation						
Ventilation type NR	126			HUI-3	0.1	Landfeldt et al.^b,c^ [23]
Adults with DMD; 96% on ventilatory support type NR	57	EQ-5D	0.44 (0.13)			Pangalila et al.^d^ [27]
		EQ-5D VAS	78 (19)			
No ventilation	NR			HUI-3	0.52 (0.03)	Landfeldt et al.^e^ [15]
Night-time ventilation	NR			HUI-3	0.13 (0.02)	Landfeldt et al.^e^ [15]
Day- and night-time ventilation	NR			HUI-3	0.05 (0.01)	Landfeldt et al.^e^ [15]
Mixed ages/status						
70% < 17 years	268	EQ-5D	0.24			Cavazza et al.^a^ [24]
		EQ-5D VAS	50.5			
Any DMD	770			HUI-3	0.46	Estimated from 17 [17]

*DMD* Duchenne muscular dystrophy, *CG* caregiver, *y* years, *NR* not reported, *SD* standard deviation, *HUI* Health Utilities Index, *EQ-5D* EuroQoL 5 dimensions, *VAS* visual analogue scale

^a^Patients were assigned to health states by the original investigators predominantly by ambulatory status; such that a non-ambulatory 10 year old would be classified as ‘early non-ambulatory’; or an ambulatory 12 year old would be classified as ‘late ambulatory’. Country-specific estimates also available (see Supplementary table); the EQ-5D scoring functions to generate country-specific estimates was not specified

^b^Estimates based on CG rating of current patient health, current mental status also available; see Supplementary table

^c^Utility for ventilated patients who are a subset of the overall [17] sample

^d^EQ-5D scoring function not specified

^e^Utilities for ventilation from Landfeldt 2015 are from the UK subset of [17]

### Caregiver utility estimates

Utility estimates from caregivers of DMD patients of mixed ages and functional status, are presented in Table 4. One EQ-5D derived utility estimate was 0.81 (EQ-5D VAS 74.0) for a mixed-age and -status cohort of DMD patients [24]. Another study by Landfeldt et al. reported caregiver utilities according to the ambulatory status and age of DMD patients, and mean (SD) EQ-5D utilities ranged from 0.85 (0.19) for caregivers of patients with early ambulatory DMD, to 0.79 (NR) for caregivers of patients with late non-ambulatory DMD [18, 23]. This reflected mean (SD) EQ-5D utility decrements of 0.09 (0.21) and 0.14 (0.29) for caregivers of patients with ambulatory and non-ambulatory DMD, respectively, compared to age- and sex-matched individuals from the general population [18]. One study reported a mean (SD) EQ-5D utility of 0.87 (0.17) for caregivers of adults with DMD, almost all of whom were on ventilatory support [27]. Less variability was observed from country-specific caregiver samples, compared to patient samples (Fig. 2). Caregiver utilities stratified by caregiver perceptions of the physical and mental health of DMD patient are presented in Supplementary Table 4.

**Table 4:** DMD caregiver utilities, by DMD patient clinical stage

Patient health state	*n*	Measure	Mean (SD) utility	Source
Ambulatory				
Early ambulatory (age 5–7 years)	155	EQ-5D	0.85 (0.19)^a^	Landfeldt et al. [18, 23]^c^
		EQ-5D VAS	76	Landfeldt et al. [18, 23]^c^
Late ambulatory (age 8–11 years)	256	EQ-5D	0.83	Landfeldt et al. [18, 23]^c^
		EQ-5D VAS	75	Landfeldt et al. [18, 23]^c^
Age- and sex-matched disutility^b^			0.09 (0.21)	Landfeldt et al. [18, 23]^c^
Non-ambulatory				
Early non-ambulatory (age 12–15 years)	154	EQ-5D	0.77 (0.03)^a^	Landfeldt et al. [18, 23]^c^
		EQ-5D VAS	71	Landfeldt et al. [18, 23]^c^
Late non-ambulatory (age 16 + years)	205	EQ-5D	0.79	Landfeldt et al. [18, 23]^c^
		EQ-5D VAS	74	Landfeldt et al. [18, 23]^c^
Age- and sex-matched disutility^b^			0.14 (0.29)	Landfeldt et al. [18, 23]^c^
On ventilatory support				
Adults with DMD’; 96% on ventilatory support	80	EQ-5D	0.87 (0.17)	Pangalila et al.^d^ [27]
		EQ-5D VAS	81 (15)	
No ventilation	NR	EQ-5D	0.84 (0.01)	Landfeldt et al.^e^ [15]
Night-time ventilation	NR	EQ-5D	0.78 (0.03)	Landfeldt et al.^e^ [15]
Day- and night-time ventilation	NR	EQ-5D	0.77 (0.03)	Landfeldt et al.^e^ [15]
Mixed ages/status				
Of DMD patients, 70% < 17 years	770	EQ-5D	0.81	Landfeldt et al.^c^ [18, 23]
	770	EQ-5D VAS	74 (0.14)^a^	
Of DMD patients, aged 8–17 years	154	EQ-5D	0.71	Cavazza et al. [24]^c^
		EQ-5D VAS	74.7	

*DMD* Duchenne muscular dystrophy, *y* years, *SD* standard deviation, *EQ-5D* EuroQoL 5 dimensions, *VAS* visual analogue scale

^a^SD estimated from 95% confidence interval

^b^Disutility across ambulatory or non-ambulatory status estimated versus the general population

^c^Country-specific estimates also available; see Supplementary table

^d^EQ-5D scoring function used not specified

## Discussion

This systematic review highlights that there are relatively few published studies evaluating utility values for DMD; only five unique studies were identified (with one study reporting related findings in four publications). While one of these was identified in a previous systematic review [16], the other citation from that prior review was not eligible here as it was from an abstract presented more than two years before the date of search [16]. Nonetheless, all available evidence of utility values for DMD—which is derived entirely from preference-based instruments—is consistent in documenting the substantial impact of DMD on HRQoL. Estimates for utility values for DMD health states ranged from 0.75 for patients with early ambulatory DMD [17], to 0.05 for later-stage patients on ventilatory support [15]. Substantially lower utility was observed among DMD patients who were non-ambulatory compared to those remaining ambulatory [15, 17, 25]. Across studies, patient utilities tended to be lower with more severe patient disease by objective criteria such as ambulatory status but also by subjective criteria like caregiver impression of patient health. While it may appear intuitive that utility would decline with the progressive, devastating functional impacts associated with DMD disease progression, the relationship is not necessarily linear. This is in part because as patients with DMD have only ever known a life of chronic illness, they would accommodate to their symptoms despite the severity of their condition. Patients with chronic diseases may thus experience changes consistent with a “re-baselining” of HRQoL as they learn to adapt to their new health state, a phenomenon known as response shift [29]. Consequently, utility values from DMD patients living longer within a non-ambulatory health state (or from their proxies) might well be higher than from patients experiencing the health impact for the first time. A full appreciation of the impact that rare pediatric diseases, such as DMD, have on HRQoL is further complicated by several factors including the small number of patients with the condition, varying inclusion criteria between studies limiting comparability, and that data are collected from proxy individuals as many HRQoL measures are not validated for self-completion by young children [30, 31].

Utility values from caregivers—which were also entirely derived using generic, preference-based instruments—were higher and showed less variability than for DMD patients; ranging from 0.85 for caregivers of early ambulatory patients to 0.83 for caregivers of late ambulatory patients [18]. In general, utility values for caregivers of non-ambulatory patients were lower—ranging from 0.77 (caregivers of patients on day and night ventilation) [15] to 0.79 (caregivers of late non-ambulatory patients) [18], with the exception of the estimate from Pangalila et al. (0.87) [27]. That study reported comparable HRQoL between caregivers of adult DMD patients and the general population; and that subjective caregiver burden did not vary depending on whether the son with DMD lived at home [27]. Given that the study by Pangalila et al. included caregivers of adult patients with DMD, adaptation to or coping with the caregiving role over the lifetime of their child could possibly help explain relatively high utility values. Other potential explanatory factors could include the satisfaction respondents report with their caregiving role, relatively low rates of anxiety and depression, and the degree of help from home attendants offered as regular support in the Netherlands [27]. Caregiver utility values were lower in the study by Landfeldt et al. [18], which assessed utility from individuals caring for younger patients with DMD, who would be living at home and would be earlier in their disease course than the Dutch adults. Thus, the burden of uncertainty regarding their child’s severity of disease course and the expectation of their future health states may contribute to lower utility values. In addition, approximately half of the caregivers in Landfeldt et al. reported at least moderate anxiety and depression, highlighting the importance of mental health status on caregiver utility.

Utility values for individual DMD health states are sparse. To date, health states in DMD have been exclusively defined based on utility data from generic preference-based instruments (such as EQ-5D or HUI), stratified by age, ambulatory status, and need for ventilation; and most individual health states had only one or two values reported. While generic preference-based instruments have been widely used, extensively validated, and offer a common metric to compare HRQoL across diseases, they may be less sensitive to the effects of illnesses that do not solely manifest as changes in functioning. For example, the impact of hope, fear, fatigue, social participation and dignity, which are all known to be dimensions of HRQoL important to people with DMD [32], may not be well-captured by generic preference-based instruments. The impact of these aspects on HRQoL may be profound given that the loss of ambulation tends to occur around the time that a DMD patient’s peers would be gaining independence. Therefore, there may be justification for the use of disease-specific preference-based instruments to capture the full range of effects that DMD can have on both patients and their caregivers [32, 33]. However, their value for decision-making has yet to be understood [34]. It is worth noting that, in contrast to the EQ-5D, the HUI-3 has greater domain coverage and directly measures ambulation (6 levels), dexterity (6 levels), pain, and discomfort (5 levels). These are all relevant aspects for patients with DMD and thus might be better suited for characterizing the broader impact of the condition on HRQoL. Utility data for other dimensions of health which may be considered additional key determinants of the HRQoL associated with DMD (e.g. loss of hand function, respiratory function decline to < 1 L forced vital capacity, mobility impacts prior to loss of ambulation, cardiomyopathy, scoliosis, developmental disability, or fatigue) [35] have not yet been characterized, with any utility instrument. Assessing utility before and after loss of ambulation in a more granular way will allow for more robust estimates of DMD specific HRQoL as the disease progresses. Following loss of ambulation, properly describing upper body functioning becomes paramount because most individuals with DMD maintain upper extremity function for a prolonged time [36]. The ability to perform activities of daily living (as self-feeding, use of computers and phones, brushing teeth, dressing, or using the toilet independently) enables non-ambulatory patients to maintain a sense of independence [37]. Therefore, thorough investigation of the impact of these discrete events on patient utility as disease progresses—either as an absolute value or as a disutility that could be applied to the underlying utility of a core DMD-related health state—would better represent patient heterogeneity and facilitate more robust cost-effectiveness modelling.

As expected, given the severe impact of DMD progression on HRQoL, reported utilities differed markedly between health states. However, even within a health state, substantial variability was observed, particularly for utilities for non-ambulatory DMD. One plausible explanation is that the health states studied include a relatively clinically heterogeneous population who experience substantially different disease. For example, within the category of ‘non-ambulatory DMD’, both patients who had recently lost ambulation (but have preserved upper limb and respiratory function), and patients with advanced DMD on full-time ventilation, could be included. When considered separately, the utility for patients on day-and-night ventilation is very low (utility = 0.05), whereas the utility of patients on nighttime ventilation is 0.13 [15], and among early non-ambulatory patients, 0.24 [17]. It is interesting to note that, aside from utility values from ventilated individuals, no other stratifications of non-ambulatory patients have been attempted when estimating utility. As previously mentioned, utility values for health states described at a more granular level would more adequately capture the nuances of non-ambulatory health.

In addition to between-patient heterogeneity, variability in health state utilities could also be introduced by other factors that differed both between studies and within health states, including respondent type, utility instrument, and sample selection criteria. As reported in numerous other conditions, patient and proxy utilities for the same health state can markedly differ; [38, 39] and indeed in DMD, patient utilities tended to be higher than those obtained from proxy respondents for the same health states [17, 24, 25, 27]. One hypothesis for this is that patients experiencing a health state adapt to the condition in a way that other respondent types cannot imagine, resulting in relatively higher utility values than estimates derived from proxy respondents [40]. In addition, the way in which individuals respond to questions about HRQoL may be influenced by the people that surround them. Patients, including children, may be cognizant of how their self-assessments of health might impact their family or caregivers, giving rise to higher utility values than those reported by proxy respondents [41]. In the studies included in the current review, given that patient and proxy utilities for the same health state were not collected within the same study, differences in study design and recruitment strategies could also contribute to observed differences. It is interesting to note that no studies were identified that elicited utility values from general population respondents using direct measurement methods (e.g. using a vignette-based approach). Nor were utility decrements (or disutilities) for clinical events occurring among DMD patients identified.

In addition to the two studies reporting country-specific estimates from the UK [15] and the Netherlands [27], two studies reporting on multi-country data collection exercises provided insight into how utility estimates can vary according to nationality. Landfeldt et al. presented HUI-3-based estimates according to age and ambulatory status from four countries, which showed similar trends of lower utility with higher DMD severity [15, 17]. EQ-5D-based estimates from Cavazza et al. showed more intercountry variability [24], potentially due to smaller sample sizes, differences in the severity of patients included across studies, and country-specific differences in the interpretation of the impact of DMD progression on HRQoL. The HUI-3 has been used among respondents from numerous countries. In general and in the studies using HUI-3 cited in this systematic review, health states are valued using the standard HUI-3 scoring function based on community preferences in Canada. Thus, unlike with the EQ-5D, the use of country-specific scoring functions for HUI-3 is not a source of heterogeneity [10]. The EQ-5D was developed using a different approach where country-specific validation studies have resulted in the creation of country-specific tariffs (value sets) used to convert EQ-5D scores into utility values; [42] and many studies using the EQ 5D have reported differences in general population utilities by country [43, 44]. While there was no explicit mention of different tariffs applied in the EQ-5D studies reviewed in this SLR, the application of such tariffs would have also contributed to the variability in country-specific estimates. Additional variability could be attributed to differences in standards of care or clinical practice between countries that could in turn impact the types and severities of patients contributing data to different health states; [16] for example, in the timing of initiation of various types of ventilatory support or scoliosis surgery,

It is notable, but not surprising, that no utility data collected within clinical trials were identified in the literature. In rare pediatric diseases such as DMD, trial-based utilities for a full set of disease-specific health states are difficult to obtain for several reasons. First, samples enrolled in trials for treatments for rare diseases are small [45], giving rise to data collection challenges for parameters like HRQoL or utility values. The impact of small samples is further magnified by the observed variability in patterns of progression and functional impairments between individuals and over time, consistent with other rare pediatric conditions [46, 47]. Second, the selection criteria, time horizon, and length of follow-up might mean that trials may not be capable of capturing information on all health states (for example, by enrolling younger healthier patients who have not yet progressed vs patients more advanced in the course of disease), and will likely vary between trials for different agents. Further, there are substantial challenges in ensuring that many pediatric patients understand and respond appropriately to HRQoL and preference measures. Most self-report instruments were designed for and validated among older children (e.g. those ≥ 7 years of age) [30, 31]. More fundamentally, patients with rare diseases are born into a life of chronic illness, so measuring self-perceived HRQoL as they adapt to their circumstances could also pose challenges [48].

Despite variability in existing estimates and the challenges inherent in measuring utilities for rare pediatric diseases, robust estimates are still required to inform value appraisals of DMD therapies. The choice of baseline utility for a model is particularly important because it affects the potential incremental gain achievable by different therapeutic options. ICER in the US, and other health technology assessment agencies globally, have published clear guidelines for utility elicitation to inform economic models [13, 49]. They specify that health state utilities should reflect the preferences of the general public, as the economic models developed for or by these agencies typically inform decisions made at the population level. Indirectly elicited HRQoL data from a generic preference-based classification instrument are generally preferred. Other strategies, such as the mapping function between the functional assessment tool (DMDSAT) and HUI-3 utilities [15] and newer DMD-specific instruments [32], hold promise for helping to identity utility estimates for wider aspects of functioning in patients with DMD. No other published data were identified in this SLR from mapping studies, which is not surprising given the lack of sensitivity and reliability of some of the most commonly used instruments (like the Pediatric Outcomes Data Collection Instrument [PODCI] and Pediatric Quality of Life Inventory [PedsQL]-generic) for DMD patient populations [25, 50, 51]. While instruments more specific to DMD exist (such as the PedsQL neuromuscular module (NMM) and DMD modules), they were established to estimate the burden of disease among DMD patients vs healthy subjects rather than for measuring changes in disease trajectory or relative *treatment* benefits. They also require a substantially sized pool of patient respondents that may be difficult to achieve in a rare disease setting [47]. Aside from using indirect, preference-based instruments, general population preferences may also be directly elicited using the SG or TTO in a vignette-based elicitation that is designed to estimate utilities for the full breadth of health states experienced by patients with a target condition. Such exercises may be particularly useful for rare diseases where the sizes of patient populations limit the number of patients who could report on their health directly. [52],. Although they have the advantage of potentially generating more sensitive disease-specific utilities, they also have challenges; including in accurately describing and valuating the HRQoL status of patients along the full continuum of DMD health states describing disease progression; that they can be conceptually difficult for respondents to understand; and that a host of unmeasured factors can impact a respondents preferences—including, for example, whether one is a parent or more familiar with the health state in question [53]. Nonetheless, as noted above, at this time no published studies were identified.

Published utilities for DMD health states characterized by age, ambulatory status, and need for ventilation document the dramatic HRQoL impact associated with the progression of DMD. However, utility values for many common health states that impact the HRQoL of patients with DMD and their caregivers are not presently available; including for non-ambulatory patients with different levels of arm or respiratory function, for example. Despite well-documented challenges in collecting HRQoL data among patients with rare diseases, further consideration of methodological options for expanding on the existing utilities for DMD is warranted. These may include larger mapping studies, prospective data collection exercises focusing on patients and their caregivers, or initiatives involving a wider variety of stakeholders such as members of the general public or clinicians. Utilities for a wider range of DMD health states will be needed to provide a more accurate representation of natural history of DMD as well as more accurate value assessment for treatments that have the potential to alter the course of DMD progression

## Electronic supplementary material

Below is the link to the electronic supplementary material.

Supplementary material 1 (PDF 179 kb)
