# Supplementary material for: Characterizing health state utilities associated with Duchenne muscular dystrophy: a systematic review
Source: Qual Life Res. 2019 Dec 6;29(3):593–605. doi: 10.1007/s11136-019-02355-x (PMC7028804; doi:10.1007/s11136-019-02355-x)
Supplement: Supplementary file 1 — Supplementary material 1 (PDF 179 kb) [file 11136_2019_2355_MOESM1_ESM.pdf]

Supplementary Table 1: Search strategy

		Search term	Results
**MEDLINE**			
Population	1	DMD.ti,ab,ot.	16648
	2	(duchenne$ adj3 (syndrome$ or morbus)).ti,ab,ot,hw.	142
	3	((duchenne$ or pseudo hypertrophic or pseudohypertrophic) adj3 dystroph$).ti,ab,ot,hw.	28315
	4	Duchenne muscular dystrophy/	19408
	5	or/1-4	32117
	6	(caregiver or caregiv* or care-giv*).ti,ab,ot,hw.	179019
	7	5 and 6	259
	8	(carer or carer* or caring).ti,ab,ot,hw.	111680
	9	5 and 8	119
	10	proxy/	2167
	11	5 and 10	5
	12	(parent or mother or father or son or family).ti,ab,ot,hw.	2559270
	13	5 and 12	2630
	14	7 or 9 or 11 or 13	2811
	15	animal/	7727104
	16	animal experiment/	2313035
	17	(rat or rats or mouse or mice or murine or rodent or rodents or hamster or hamsters or pig or pigs or porcine or rabbit or rabbits or animal or animals or dogs or dog or cats or cow or bovine or sheep or ovine or monkey or monkeys).ti,ab,ot,hw.	13202997
	18	or/15-17	13202997
	19	14 not 18	2498
	20	5 not 18	22414
Outcomes	21	"Value of Life"/	129109
	22	quality of life/	581879
	23	quality adjusted life/	0
	24	quality adjusted life year/	33619
	25	quality of life index/	2552
	26	short form 6/ or short form 36/	24512
	27	sickness impact profile/	9232
	28	(quality adj2 (wellbeing or well-being)).ti,ab.	3948
	29	(quality of wellbeing or quality of well being or index of wellbeing or index of well being or qwb).ti,ab.	1190
	30	sickness impact profile.ti,ab.	2239
	31	disability adjusted life.ti,ab.	6048

	32	(qal* or qtime* or qwb* or daly* or qtime* or life year or life years).ti,ab.	48476
	33	(euroqol* or eq5d* or eq 5d*).ti,ab.	25792
	34	(qol* or hql* or hqol* or h qol* or hrqol* or hr qol*).ti,ab.	129374
	35	(pqol or qls).ti,ab.	937
	36	(health utility* or utility score* or disutilit*).ti,ab.	6418
	37	(hui or hui1 or hui2 or hui3).ti,ab.	3329
	38	health* year* equivalent*.ti,ab.	80
	39	(health* adj2 year* adj2 equivalent*).ti,ab.	99
	40	(health adj3 (utilit* or status)).ti,ab.	145359
	41	(utilit* adj3 (valu* or measur* or health or life or estimat* or elicit* or disease or score* or weight)).ti,ab.	27486
	42	(preference* adj3 (valu* or measur* or health or life or estimat* or elicit* or disease or score* or instrument or instruments)).ti,ab.	21552
	43	rosser.ti,ab.	200
	44	(hye or hyes).ti,ab.	183
	45	(willingness to pay or time tradeoff or time trade off or tto or standard gamble*).ti,ab.	16748
	46	(sf36 or sf 36 or short form 36 or shortform 36 or short form36 or shortform36 or sf thirtysix or sfthirtysix or sfthirty six or sf thirty six or shortform thirtysix or shortform thirty six or short form thirtysix or short form thirty six).ti,ab.	60639
	47	(sf6 or sf 6 or short form 6 or shortform 6 or sf six or sfsix or shortform six or short form six or shortform6 or short form6).ti,ab.	4005
	48	(sf6D or sf 6D or short form 6D or shortform 6D or sf sixD or sfsixD or shortform sixD or short form sixD or shortform6D or short form6D).ti,ab.	2097
	49	nottingham health profile*.ti,ab.	2594
	50	sickness impact profile.ti,ab.	2239
	51	exp health status indicators/	303835
	52	duke health profile.ti,ab.	193
	53	functional status questionnaire.ti,ab.	277
	54	dartmouth coop functional health assessment*.ti,ab.	23
	55	or/21-54	1216892
DMD carers and outcomes	56	19 and 55	277
DMD and outcomes	57	20 and 55	1042
**EMBASE**			
Population	58	56 use oemezd	191
	59	57 use oemezd	698

	60	DMD.ti,ab,ot.	16648
	61	(duchenne$ adj3 (syndrome$ or morbus)).ti,ab,ot,hw.	142
	62	((duchenne$ or pseudo hypertrophic or pseudohypertrophic) adj3 dystroph$).ti,ab,ot,hw.	28315
	63	Muscular Dystrophy, Duchenne/	12810
	64	or/60-63	32117
	65	(caregiver or caregiv* or care-giv*).ti,ab,or,hw.	179017
	66	64 and 65	259
	67	(carer or carer* or caring).ti,ab,ot,hw.	111680
	68	64 and 67	119
	69	proxy/	2167
	70	64 and 69	5
	71	(parent or mother or father or son or family).ti,ab,ot,hw.	2559270
	72	64 and 71	2630
	73	66 or 68 or 70 or 72	2811
	74	exp animals/ not (exp animals/ and humans/)	16132559
	75	73 not 74	2057
	76	64 not 74	20830
	77	"Value of Life"/	129109
	78	quality of life/	581879
	79	quality adjusted life/	0
	80	quality-adjusted life years/	33619
	81	sickness impact profile/	9232
	82	(quality adj2 (wellbeing or well-being)).ti,ab.	3948
	83	(quality of wellbeing or quality of well being or index of wellbeing or index of well being or qwb).ti,ab.	1190
	84	sickness impact profile.ti,ab.	2239
	85	disability adjusted life.ti,ab.	6048
	86	(qal* or qtime* or qwb* or daly*).ti,ab.	33298
	87	(euroqol* or eq5d* or eq 5d*).ti,ab.	25792
	88	(qol* or hql* or hqol* or h qol* or hrqol* or hr qol*).ti,ab.	129374
	89	(pqol or qls).ti,ab.	937
	90	(health utility* or utility score* or disutilit*).ti,ab.	6418
	91	(hui or hui1 or hui2 or hui3).ti,ab.	3329
	92	health* year* equivalent*.ti,ab.	80
	93	(health* adj2 year* adj2 equivalent*).ti,ab.	99
	94	(health adj3 (utilit* or status)).ti,ab.	145359
Outcomes	95	(utilit* adj3 (valu* or measur* or health or life or estimat* or elicit* or disease or score* or weight)).ti,ab.	27486

	96	(preference* adj3 (valu* or measur* or health or life or estimat* or elicit* or disease or score* or instrument or instruments)).ti,ab.	21552
	97	rosser.ti,ab.	200
	98	(hye or hyes).ti,ab.	183
	99	(willingness to pay or time tradeoff or time trade off or tto or standard gamble*).ti,ab.	16748
	100	(sf36 or sf 36 or short form 36 or shortform 36 or short form36 or shortform36 or sf thirtysix or sfthirtysix or sfthirty six or sf thirty six or shortform thirtysix or shortform thirty six or short form thirtysix or short form thirty six).ti,ab.	60639
	101	(sf6 or sf 6 or short form 6 or shortform 6 or sf six or sfsix or shortform six or short form six or shortform6 or short form6).ti,ab.	4005
	102	(sf6D or sf 6D or short form 6D or shortform 6D or sf sixD or sfsixD or shortform sixD or short form sixD or shortform6D or short form6D).ti,ab.	2097
	103	nottingham health profile*.ti,ab.	2594
	104	sickness impact profile.ti,ab.	2239
	105	exp health status indicators/	303835
	106	duke health profile.ti,ab.	193
	107	functional status questionnaire.ti,ab.	277
	108	dartmouth coop functional health assessment*.ti,ab.	23
	109	or/77-108	1207845
DMD carers and outcomes	110	75 and 109	167
DMD and outcomes	111	76 and 109	699
MEDLINE	112	110 use ppezv	87
MEDLINE	113	111 use ppezv	356
	114	112 or 56	279
	115	113 or 57	1056
**(DMD + CG terms) + OUTCOMES**	**116**	**remove duplicates from 114**	222
**All DMD terms + OUTCOMES**	**117**	**remove duplicates from 115**	888

Supplementary Table 2: Study quality assessment

Within the study quality assessment, one point was awarded for each of the following criteria: (1) sample size ≥100; (2) description of respondent selection and recruitment; (3) description of inclusion/exclusion criteria; (4) response rate ≥ 60%; (5) reporting of attrition/loss to follow-up (for longitudinal studies only); (6) reporting of missingness of data and approaches to deal with it; (7) appropriateness of measure (based on the authors' judgment). Lastly, the scores were summed for each article to yield an overall quality score, ranging from 0 to 7 (for longitudinal studies) or 0 to 6 (for cross-sectional studies) where higher scores indicated higher quality.[26]

Citation	n	Recruitment	IC/EC	Response	LFU	Missing data	Measure	Total Score
Cavazza et al, 2016 [29]	1	1	1	0	C	1	1	5/6
Landfeldt et al, 2014, 2015, 2016 [15; 17; 27; 28]	1	1	1	0	C	1	1	5/6
Landfeldt et al, 2018 [30]	1	1	1	0	C	0	1	4/6
Pangalila et al, 2011 [32]	1	1	1	0	C	1	1	5/6

C = Cross-sectional study, criterion not applicable

n=Appropriate sample size; Recruitment= Appropriate and robust respondent recruitment strategy; IC/EC=Inclusion/exclusion criteria clearly specified; Response=Response rates reported; LFU=Loss to follow-up reported; Missing data=Amount and nature of missing data described; Measure=Measure appropriateness

The quality of the utilities included in Magnetta et al.,[31] could not be rated due to that only few details were reported.

Supplementary Table 3: Utilities for DMD patients with heart failure [31]

	Mean utility
NYHA class I	0.7
NYHA class II	0.63
NYHA class III	0.55
NYHA class IV	0.44

NYHA: New York Heart Association

Supplementary Table 4: Stratified utility values for (top) DMD patients; and (bottom) DMD caregivers [27; 28]

Health state	n	Mean HUI-3 utility
Caregiver-rated patient physical health		
Excellent	145	0.62
Very good	321	0.5
Good	228	0.37
Fair	70	0.22
Poor	6	0
Caregiver-rated patient mental health		
Happy/interested	455	0.56
Somewhat happy	239	0.36
Somewhat unhappy	63	0.22
Very unhappy	13	0.04

Health state	n	Mean EQ-5D utility
Caregiver-rated patient physical health		
Excellent	145	0.88 (0.15)
Very good	321	0.83
Good	228	0.77
Fair/poor	76	0.71
Caregiver-rated patient mental health		
Happy/interested	455	0.84
Somewhat happy	239	0.79
Somewhat unhappy	63	0.69
Very unhappy	13	0.57 (0.32)

DMD: Duchenne muscular dystrophy; HUI: Health Utilities Index
